# Th17-Related Genes and Celiac Disease Susceptibility

**DOI:** 10.1371/journal.pone.0031244

**Published:** 2012-02-16

**Authors:** Luz María Medrano, Manuel García-Magariños, Bárbara Dema, Laura Espino, Carlos Maluenda, Isabel Polanco, M. Ángeles Figueredo, Miguel Fernández-Arquero, Concepción Núñez

**Affiliations:** 1 UGC de Inmunología, Hospital Clínico San Carlos, Instituto de Investigación Sanitaria del Hospital Clínico San Carlos (IdISSC), Madrid, Spain; 2 Unidade de Xenética, Instituto de Medicina Legal and Departamento de Anatomía Patológica y Ciencias Forenses, Facultade de Medicina, Universidade de Santiago de Compostela, Santiago de Compostela, Spain; 3 Departamento de Estadística e IO, Universidad Pública de Navarra, Pamplona, Spain; 4 Servicio de Pediatría, Hospital Clínico San Carlos, Madrid, Spain; 5 Servicio de Gastroenterología Pediátrica, Hospital La Paz, Madrid, Spain; Emory University School of Medicine, United States of America

## Abstract

Th17 cells are known to be involved in several autoimmune or inflammatory diseases. In celiac disease (CD), recent studies suggest an implication of those cells in disease pathogenesis. We aimed at studying the role of genes relevant for the Th17 immune response in CD susceptibility. A total of 101 single nucleotide polymorphisms (SNPs), mainly selected to cover most of the variability present in 16 Th17-related genes (*IL23R, RORC*, *IL6R*, *IL17A*, *IL17F*, *CCR6*, *IL6*, *JAK2*, *TNFSF15*, *IL23A*, *IL22*, *STAT3*, *TBX21*, *SOCS3*, *IL12RB1* and *IL17RA*), were genotyped in 735 CD patients and 549 ethnically matched healthy controls. Case-control comparisons for each SNP and for the haplotypes resulting from the SNPs studied in each gene were performed using chi-square tests. Gene-gene interactions were also evaluated following different methodological approaches. No significant results emerged after performing the appropriate statistical corrections. Our results seem to discard a relevant role of Th17 cells on CD risk.

## Introduction

Celiac disease (CD) is an immune related disease mainly characterized by intestinal inflammation after gluten ingestion in genetically susceptible individuals. CD has been traditionally considered a Th1-mediated disease. However, accumulating evidence about the relevant role of the novel Th17 immune response in several autoimmune diseases [1] opened the possibility towards an involvement of this immunological pathway in CD pathogenesis. These cells seem to be involved in protective responses against extracellular pathogens but they can contribute to chronic inflammation and autoimmunity when dysregulated.

Th17 cells develop from naïve CD161+ CD4+ T cells upon stimulation with particular immunological stimulus, especifically, transforming growth factor beta (TGF-β), interleukin (IL)-23, IL-1β or IL-6 [2]. This induces several transcription factors, mainly the RAR-related orphan receptor C (RORC), which in turn activates IL-17A and IL-17F transcription, the distinctive effector cytokines of this subset of T cells. Production of IL-21, IL-22 and IL-26 also characterizes this specific response, besides the surface markers C-C chemokine receptor type 6 (CCR6) and IL-23 receptor (IL-23R).

Studies based on murine models of several autoimmune diseases, as multiple sclerosis (autoimmune encephalomyelitis, EAE), rheumatoid arthritis (collagen-induced arthritis, CIA) and inflammatory bowel disease (experimental colitis), provided the first evidence about a role of Th17 cells in those conditions [3], [4]. This idea was later supported by case-control studies, which associated genetic variants in *IL23R* with susceptibility to Crohn's disease, psoriasis and ankylosing spondylitis [5], [6], [7]. Nowadays, the Th17 immune response is considered as a relevant player in several autoimmune or inflammatory diseases. IL-17 mRNA or protein have been detected in biological fluids or the specific affected tissue in several autoimmune disorders [8] and genetic studies associated genes coding important Th17 related products with several diseases [9]. In addition, epistasis between *IL23R* and other Th17 related genes has been reported: with *IL2/IL21* in UC [10] and with *IL17A* and *IL17RA* in Crohn's disease [11].

In 2008, a putative implication of the Th17 immune response in CD pathogenesis was suggested from two studies following different approaches. Our research group detected a significant association between a genetic polymorphism in the *IL23R* gene and CD [12] and Harris et al. found higher production of IL-23 after stimulation of human monocytes derived from CD patients with peptic fragments of wheat gliadin [13]. Subsequently, genetic linkage with the *IL23R* region was observed in Finnish families, although this result was not replicated in Hungarian pedigrees and no association with *IL23R* polymorphisms was observed in Finnish, Hungarian or Italian CD samples [14]. In addition, increased expression of several Th17-related cytokines or products was detected in patients with active CD [15], [16] and very recently, gluten-specific IL-17A-producing cells have been found in the duodenum of CD patients [17], which supports a role of Th17 cells in CD pathogenesis.

Despite these results observed in CD, the role of the Th17 cells on this disease is still not well defined. We aimed at shedding more light upon this issue by performing an extensive genetic study including many genes coding distinctive cytokines, markers or transcription factors involved in the Th17 response. We will evaluate the individual influence of those genes on CD susceptibility and also the possible contribution of gene-gene interactions. Previous genome wide association studies (GWAS) did not find association with CD susceptibility of any Th17-related gene [18], [19], [20] (with exception of the *IL2/IL21* locus, also involved in other processes), but we consider that a different scenario could emerge with this study: we cover most of the variability present in the studied genetic regions and we will evaluate the genetic interactions between the included polymorphisms, which has been proved as a valid approach to detect new susceptibility variants 21,22.

## Materials and Methods

### Ethics Statement

This study was approved by the ethical committee (CEIC) of the Hospital Clínico San Carlos. Samples were obtained after obtaining written informed consent.

### Subjects

A total of 735 CD patients and 549 ethnically matched healthy controls were included in the initial study. A second sample set consisting of 294 CD patients and 475 controls was used for additional analysis. All these samples correspond to unrelated Spanish white individuals. CD patients were diagnosed following the European Society for Paediatric Gastroenterology and Nutrition (ESPGAN), 97% are positive for HLA-DQ2 and/or HLA-DQ8. Controls correspond mainly to blood donors and laboratory staff. CD samples were consecutively collected in two centres of the same region (Hospital La Paz and Hospital Clínico San Carlos, Madrid) and controls were collected at the Hospital Clínico San Carlos.

### Markers and genotyping

We selected genes with a known functional role in the Th17 immune response. Accordingly, sixteen genes were studied: *IL23R*, *RORC*, *IL6R*, *IL17A*, *IL17F*, *CCR6*, *IL6*, *JAK2*, *TNFSF15*, *IL23A*, *IL22*, *STAT3*, *TBX21*, *SOCS3*, *IL12RB1* and *IL17RA*. For all these genes except *IL6R*, *JAK2* and *STAT3*, single nucleotide polymorphisms (SNPs) were selected following the “aggressive tagging” option present in the Haploview program with genetic data downloaded from the HapMap Project (http://hapmap.ncbi.nlm.nih.gov) (50 kb upstream and downstream of the transcription initiation site). To increase statistical power, only markers with a minor allele frequency (MAF)>10% were included. In addition, SNPs located in those genes which code nonsynonymous changes or were previously associated with some autoimmune disease were also analysed independently of their MAF. In *STAT3* and *JAK2*, only two SNPs previously associated with Crohn's disease, which share some susceptibility factors with CD, were included: rs744166 and rs10758669, respectively; and in *IL6R* we studied one functional polymorphism, rs8192284. SNPs located in *IL6* and *IL6R* and two SNPs in *IL23R*, rs11209026 and rs7517847, were analysed in previous works [12], [23], which included most of the samples initially analysed in this study, but their data were used to evaluate genetic interactions with other Th17 related genes.

A total of 101 SNPs were initially studied (Table S1). All of them were genotyped by Veracode technology performed at the National Genotyping Center (http://www.cegen.org), except those that failed (rs10494269, rs9395767, rs608137, rs6927645, rs273506 and rs2241044) and those located in the *IL6*, *IL6R* and *TBX21* genes, which were genotyped with specific TaqMan assays. Two SNPs (rs11209026 and rs7517847, both in the *IL23R* gene) were genotyped by those two technologies and identical results were obtained.

Additional analysis included the study by TaqMan technology of rs12070470, in the *IL23R* gene.

### Statistical analysis

Deviations from Hardy-Weinberg proportions were assessed in all the SNPs studied.

A case-control analysis using chi-square tests was performed for each individual SNP and for the haplotypes resulting from the SNPs studied in the same genetic region.

Interactions between genes were evaluated following four different approaches: logistic regression, random forests (RF), classification and regression trees (CART) and multifactor dimensionality reduction (MDR).

## Results

Three SNPs showed deviation from Hardy-Weinberg proportions and were eliminated from the study: rs2064331 (*IL17F*), rs10878804 (*IL22*) and rs9645406 (*RORC*).

The comparison of genotypic frequencies between cases and controls for all the SNPs analysed achieved a nominal significant value in twelve polymorphisms located in eight different genetic regions (Table 1). Although none of them withstand Bonferroni correction, we tried to replicate associations involving *SOCS3* and *IL23R* using a second sample set. These two genes show the lowest case-control p-values in the present analysis and additionally some SNP in those genes showed a nominal significance in previous CD GWAS [20].

**Table 1:** Genetic polymorphisms which showed a nominal significant value after case-control comparisons (in decreasing significance).

GENE	SNP	GENOTYPE	p	OR	95% CI
*SOCS3*	rs4969170	AA	0.0018	0.59	0.42–0.84
*IL23R*	rs7528924	GG	0.0057	2.11	1.19–3.74
*TNFSF15*	rs17219926	CC	0.0103	1.43	1.08–1.89
*IL6*	rs2069827	GT+TT	0.016	1.51	1.06–2.14
*IL22*	rs11611206	AA	0.019	0.39	0.16–0.93
*IL23R*	rs11209026	AG+GG	0.026	1.42	1.03–1.97
*RORC*	rs1521186	AA+AG	0.027	1.31	1.02–1.67
*IL22*	rs11177131	CT+TT	0.034	0.76	0.58–0.99
*IL17A*	rs8193036	CT+TT	0.034	0.60	0.36–0.99
*IL6*	rs1800795	CG+CC	0.037	1.26	1.01–1.58
*TNFSF15*	rs6478108	CT+CC	0.043	0.79	0.63–1.00
*CCR6*	rs3798315	TT	0.044	4.17	0.90–38.84

ORs are referred to the mutant genotype or carrier of the mutant allele (specified below “genotype”).

The initial *IL23R* data analysis also evidenced one haplotype significantly associated with CD susceptibility (rs4655683-rs10889667-rs1569922-rs790632-rs7517847-rs10489629-rs7528924-rs2201841-rs4655530-rs11209026-rs6682033-rs6693831, G-C-C-C-T-A-G-T-A-G-A-C): 9.2% in CD patients vs. 6.3% in controls (p = 0.0067). For replication purposes, the SNP rs12070470, highly correlated with that haplotype (r^2^ = 1 according to http://hapmap.ncbi.nlm.nih.gov/) was studied in the second sample set instead of the 12 SNPs initially considered.

No significant associations involving *IL23R* were observed in the replication set. Regarding the SNP rs4969170, in the *SOCS3* gene, a significant association was observed pooling the original and the replication sets: p = 0.0012 OR = 0.64 95% CI 0.49–0.84 (Table 2). Statistical power limitations probably precluded us to obtain a significant result in the replication set.

**Table 2:** Genotypic data (N (%)) for rs4969170 in the original and the replication sets.

	Original set*		Replication set#	
	CD	Controls	CD	Controls
	(N = 732)	(N = 551)	(N = 294)	(N = 462)
GG	292 (39.9)	212 (38.5)	124 (42.2)	198 (42.9)
AG	368 (50.3)	253 (45.9)	138 (46.9)	199 (43.1)
AA	72 (9.8)	86 (15.6)	32 (10.9)	65 (14.1)

AA genotype:

*: p = 0.0018 OR = 0.59 95% CI 0.42–0.84;

# , p = 0.20 95% CI OR = 0.75 (0.46–1.20).

No consensus exists as to the best methodology to evaluate epistasis. Therefore, we used four different statistical methods to evaluate genetic interactions between all the studied polymorphisms located in different genes. We did not find statistically significant results with any methodological approach.

## Discussion

With the development of genome wide association studies (GWAS), the number of discovered genes involved in CD susceptibility has highly increased. However, the percentage of disease heritability explained has not experienced such an increase. Genetic variants not included in GWAS and genetic interactions could be underlying some missing heritability. We bear this in mind when studying the relevance of the Th17 immune response on CD susceptibility. We performed an extensive case-control study including sixteen genes which code relevant factors involved in that immune response. Tag SNPs were selected to cover most of the variability present in each gene, with exception of *IL6R*, *STAT3* and *JAK2*. SNPs coding nonsynonymous changes or those previously associated with other autoimmune diseases were also included in order to increase the *a priori* probability of obtaining a significant result. Additionally, we evaluated the possibility that interactions between the studied genes were involved in disease susceptibility. Our results seem to discard a relevant role of Th17 cells on CD risk, since no significantly associated SNP or gene-gene interaction was consistently observed, with the only exception of rs4969170, located in *SOCS3*, which deserves further research. However, although *SOCS3* is later confirmed, its functional role must be elucidated, since it is involved in different functional pathways and it would be expected that more than one Th17 gene was associated with CD susceptibility, as it has been observed with other Th17-mediated diseases.

The discovery of the IL-23 cytokine prompted the re-examination of the dominant Th response in many autoimmune diseases, primarily in those considered as skewed towards a Th1 phenotype. Studies based on murine models of multiple sclerosis, rheumatoid arthritis and inflammatory bowel disease related these conditions with a Th17 response. However, a similar conclusion is not drawn from GWAS results [24], [25], [26]. Although several Th17-related genes have been associated with IBD and RA, the list of MS susceptibility genes does not suggest a Th17 related etiology. This intriguing issue is probably far away from being answered. Nowadays it seems clear that Th immune responses are not independent and plasticity exists between Th cell subsets. A shift between Th1 and Th17 can occur during the inflammatory process and it is possible to speculate that the relative contribution and the timing of each subset will determine which genes would be involved in disease risk. Moreover, the cytokine microenvironment can determine the shift towards a specific immune response. From this point of view, genetics could not be so relevant if other compensatory mechanisms exist. This evidence, as previously suggested, that overlap between autoimmune diseases must be observed with caution. Th17 cells seem to mediate several autoimmune diseases but their impact in disease etiology seems to be different.

In summary, gene expression studies link CD pathogenesis to Th17 cells, but we evidenced that polymorphisms in Th17-related genes do not seem to be crucial for disease development. This is concordant with observations on MS. Although, in general, genetic data provide clues that ratified by functional studies unravel disease pathogenesis, this time it makes necessary to do somehow the other way around, with the special difficulty of explaining the divergent genetic results observed in different immune mediated diseases. Therefore, much more work is expected in this field.

## Supporting Information

Table S1 Genes and SNPs studied ordered by chromosome and position.
(DOC)
