# Supplementary material for: Th17-Related Genes and Celiac Disease Susceptibility
Source: PLoS One. 2012 Feb 16;7(2):e31244. doi: 10.1371/journal.pone.0031244 (PMC3281077; doi:10.1371/journal.pone.0031244)
Supplement: Table S1 — Genes and SNPs studied ordered by chromosome and position. (DOC) [file pone.0031244.s001.doc]

**Table S1**. Genes and SNPs studied ordered by chromosome and position.

| **GENE** | **SNP** | **CHROMOSOME** | **POSITION** |  |
| --- | --- | --- | --- | --- |
| *IL23R* | rs4655683 | 1 | 67384201 |  |
| *IL23R* | rs10889667 | 1 | 67427795 |  |
| *IL23R* | rs1569922 | 1 | 67437551 |  |
| *IL23R* | rs790632 | 1 | 67451510 |  |
| *IL23R* | rs7517847 | 1 | 67454257 |  |
| *IL23R* | rs10489629 | 1 | 67460937 |  |
| *IL23R* | rs7528924 | 1 | 67461624 |  |
| *IL23R* | rs2201841 | 1 | 67466790 |  |
| *IL23R* | rs4655530 | 1 | 67476319 |  |
| *IL23R* | rs11209026 | 1 | 67478546 |  |
| *IL23R* | rs6682033 | 1 | 67481258 |  |
| *IL23R* | rs6693831 | 1 | 67493455 |  |
| *RORC* | rs3828057 | 1 | 150046801 |  |
| *RORC* | rs12045886 | 1 | 150048714 |  |
| *RORC* | rs10494269 | 1 | 150048958 |  |
| *RORC* | rs939595 | 1 | 150050312 |  |
| *RORC* | rs1521186 | 1 | 150051171 |  |
| *RORC* | rs6693413 | 1 | 150058934 |  |
| *RORC* | rs949969 | 1 | 150059438 |  |
| *RORC* | rs4845604 | 1 | 150068304 |  |
| *RORC* | rs17582155 | 1 | 150070837 |  |
| *RORC* | rs6587622 | 1 | 150081029 |  |
| *RORC* | rs9645406 | 1 | 150083696 |  |
| *RORC* | rs16833584 | 1 | 150083858 |  |
| *IL6R* | rs8192284 | 1 | 152693594 |  |
| *IL17A* | rs13208597 | 6 | 52152468 |  |
| *IL17A* | rs9395767 | 6 | 52154729 |  |
| *IL17A* | rs4711998 | 6 | 52158312 |  |
| *IL17A* | rs8193036 | 6 | 52158452 |  |
| *IL17A* | rs2275913 | 6 | 52158992 |  |
| *IL17A* | rs7747909 | 6 | 52162208 |  |
| *IL17A* | rs1974226 | 6 | 52163294 |  |
| *IL17A/IL17F* | rs4715287 | 6 | 52174606 |  |
| *IL17A/IL17F* | rs12528203 | 6 | 52175294 |  |
| *IL17F* | rs2064331 | 6 | 52206327 |  |
| *IL17F* | rs13209590 | 6 | 52207610 |  |
| *IL17F* | rs641701 | 6 | 52209124 |  |
| *IL17F* | rs11465553 | 6 | 52209717 |  |
| *IL17F* | rs7771466 | 6 | 52212068 |  |
| *IL17F* | rs12201582 | 6 | 52212648 |  |
| *IL17F* | rs608137 | 6 | 52220675 |  |
| *CCR6* | rs2301436 | 6 | 167357978 |  |
| *CCR6* | rs150110 | 6 | 167431756 |  |
| *CCR6* | rs9364891 | 6 | 167436309 |  |
| *CCR6* | rs6927645 | 6 | 167450292 |  |
| *CCR6* | rs3798315 | 6 | 167464699 |  |
| *CCR6* | rs3093012 | 6 | 167468271 |  |
| *CCR6* | rs3093010 | 6 | 167468597 |  |
| *CCR6* | rs3093007 | 6 | 167469765 |  |
| *CCR6* | rs17860852 | 6 | 167470814 |  |
| *CCR6* | rs4710187 | 6 | 167475082 |  |
| *CCR6* | rs4710189 | 6 | 167476314 |  |
| *CCR6* | rs11575089 | 6 | 167477189 |  |
| *IL6* | rs2069827 | 7 | 22731981 |  |
| *IL6* | rs1800795 | 7 | 22733170 |  |
| *IL6* | rs2069840 | 7 | 22735097 |  |
| *JAK2* | rs10758669 | 9 | 4971602 |  |
| *TNFSF15* | rs6478106 | 9 | 116585487 |  |
| *TNFSF15* | rs6478108 | 9 | 116598524 |  |
| *TNFSF15* | rs7865494 | 9 | 116616300 |  |
| *TNFSF15* | rs17219926 | 9 | 116619674 |  |
| *TNFSF15* | rs1322063 | 9 | 116625303 |  |
| *IL23A* | rs11613055 | 12 | 55011186 |  |
| *IL23A* | rs11171806 | 12 | 55019798 |  |
| *IL22* | rs741346 | 12 | 66917607 |  |
| *IL22* | rs10878796 | 12 | 66921463 |  |
| *IL22* | rs11177131 | 12 | 66923715 |  |
| *IL22* | rs1182844 | 12 | 66927799 |  |
| *IL22* | rs976748 | 12 | 66929861 |  |
| *IL22* | rs12100005 | 12 | 66940909 |  |
| *IL22* | rs11611206 | 12 | 66954713 |  |
| *IL22* | rs10878804 | 12 | 66955524 |  |
| *IL22* | rs1383119 | 12 | 66958414 |  |
|  | rs13377617 | 12 | 67004760 |  |
| *STAT3* | rs744166 | 17 | 37767727 |  |
| *TBX21* | rs10514934 | 17 | 43167123 |  |
| *TBX21* | rs11657388 | 17 | 43175706 |  |
| *TBX21* | rs4794067 | 17 | 43163827 |  |
| *SOCS3* | rs4789588 | 17 | 73844676 |  |
| *SOCS3* | rs6501199 | 17 | 73846381 |  |
| *SOCS3* | rs11077357 | 17 | 73855196 |  |
| *SOCS3* | rs8069976 | 17 | 73861445 |  |
| *SOCS3* | rs4969170 | 17 | 73872133 |  |
| *SOCS3* | rs9900564 | 17 | 73889053 |  |
| *SOCS3* | rs4366775 | 17 | 73893674 |  |
| *IL12RB1* | rs436857 | 19 | 18058635 |  |
| *IL12RB1* | rs1870063 | 19 | 18031962 |  |
| *IL12RB1* | rs3761041 | 19 | 18044917 |  |
| *IL12RB1* | rs429774 | 19 | 18047752 |  |
| *IL12RB1* | rs374326 | 19 | 18062266 |  |
| *IL12RB1* | rs273506 | 19 | 18082647 |  |
| *IL17RA* | rs5994155 | 22 | 15929526 |  |
| *IL17RA* | rs9606603 | 22 | 15941513 |  |
| *IL17RA* | rs5748864 | 22 | 15952941 |  |
| *IL17RA* | rs13053889 | 22 | 15960394 |  |
| *IL17RA* | rs9606615 | 22 | 15960813 |  |
| *IL17RA* | rs2241044 | 22 | 15961838 |  |
| *IL17RA* | rs721930 | 22 | 15965808 |  |
| *IL17RA* | rs2241049 | 22 | 15967680 |  |
| *IL17RA* | rs879575 | 22 | 15969567 |  |
| *IL17RA* | rs2895332 | 22 | 15971089 |  |
